# Identification of four novel phosphorylation sites in estrogen receptor α: impact on receptor-dependent gene expression and phosphorylation by protein kinase CK2

**DOI:** 10.1186/1471-2091-10-36

**Published:** 2009-12-31

**Authors:** Christopher C Williams, Aninda Basu, Abeer El-Gharbawy, Latonya M Carrier, Carolyn L Smith, Brian G Rowan

**Affiliations:** 1 Division of Basic Pharmaceutical Sciences, College of Pharmacy, Xavier University of Louisiana, New Orleans, LA, USA; 2 Department of Structural and Cellular Biology, Tulane University School of Medicine, New Orleans, LA, USA; 3 Department of Molecular and Cellular Biology, Baylor College of Medicine, Houston, TX, USA

## Abstract

**Background:**

Estrogen receptor α (ERα) phosphorylation is important for estrogen-dependent transcription of ER-dependent genes, ligand-independent receptor activation and endocrine therapy response in breast cancer. However ERα phosphorylation at the previously identified sites does not fully account for these receptor functions. To determine if additional ERα phosphorylation sites exist, COS-1 cells expressing human ERα were labeled with [^32^P]H_3_PO_4 _*in vivo *and ERα tryptic phosphopeptides were isolated to identify phosphorylation sites.

**Results:**

Previously uncharacterized phosphorylation sites at serines 46/47, 282, 294, and 559 were identified by manual Edman degradation and phosphoamino acid analysis and confirmed by mutagenesis and phospho-specific antibodies. Antibodies detected phosphorylation of endogenous ERα in MCF-7, MCF-7-LCC2, and Ishikawa cancer cell lines by immunoblot. Mutation of Ser-282 and Ser-559 to alanine (S282A, S559A) resulted in ligand independent activation of ERα as determined by both ERE-driven reporter gene assays and endogenous pS2 gene expression in transiently transfected HeLa cells. Mutation of Ser-46/47 or Ser-294 to alanine markedly reduced estradiol dependent reporter activation. Additionally protein kinase CK2 was identified as a kinase that phosphorylated ERα at S282 and S559 using motif analysis, *in vitro *kinase assays, and incubation of cells with CK2 kinase inhibitor.

**Conclusion:**

These novel ERα phosphorylation sites represent new means for modulation of ERα activity. S559 represents the first phosphorylation site identified in the extreme C-terminus (F domain) of a steroid receptor.

## Background

ERα is a member of the nuclear receptor superfamily of transcription factors whose activity is primarily regulated by the binding of small lipophilic ligands. Estradiol-induced ERα signaling is indispensable for many physiological processes including reproductive tissue development (uterus, mammary gland, and ovary), bone metabolism, and immune, cardiovascular, and neurological function(1-3). Importantly, ERα has remained the primary pharmacological target for endocrine therapy of ERα positive breast cancer. Selective estrogen receptor modulators (SERMs) such as tamoxifen, as well as estrogen ablation are front line therapies for the treatment of ERα-expressing breast neoplasias.

**Figure 1:**
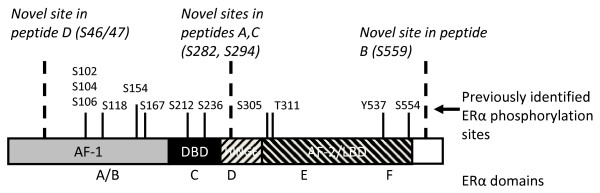
**Estrogen receptor α (ERα) phosphorylation sites**. The schematic in Figure 1 depicts both previously identified and novel ERα phosphorylation sites with relative locations within the ERα functional domains. Serines 104, 106, 118, and 167 constitute phosphorylation sites within the ligand-independent activation function-1 (AF-1) domain of ERα. S236 is the first phosphorylation site within the DNA binding domain of ERα. Serine 305, threonine 311 and tyrosine 537 are phosphorylation sites identified within the ligand-dependent activation function-2 (AF-2) domain. Indicated in ***bold italicized ***type are newly characterized phosphorylation sites of ERα: S46/47, S282, S294 and S559. S46/47 constitutes an additional site of phosphorylation within the AF-1 domain. Serines 282 and 294 are located in the hinge domain of ERα proximal to the DNA binding domain. Of note, S559 is the first phosphorylation site identified in the extreme C-terminal F domain of ERα and other steroid receptors. S154, S212, S294, S554, and S559 have been recently identified or independently confirmed by mass spectrophotometry (11).

Various aspects of ERα transcriptional activation are dependent on phosphorylation of the receptor. Coactivator recruitment, subcellular localization, receptor dimerization, ligand binding, and posttranslational modifications are regulated through the phosphorylation of individual sites of ERα. Nine ERα phosphorylation sites have been functionally characterized to date: serines 102 (S102), 104 (S104), 106 (S106), 118 (S118), and 167 (S167) in the AF-1 domain; serine 236 (S236) in the DNA binding domain; and serines 305 (S305), threonine 311 (T311), and tyrosine 537 (Y537) in the AF-2/ligand binding domain (LBD) (Figure 1). The functional interaction of ERα with coregulator proteins such as CBP/p300 and the p160 family of coactivators is regulated by phosphorylation of ERα in the AF-1 domain [1-4]. S118 is phosphorylated in response to both estradiol and epidermal growth factor through CDK7 and ERK1/2 dependent pathways, respectively [5,6]. Phosphorylation of S118 in conjunction with S104 and S106 mediates ligand independent activation of ERα by facilitating functional ERα interactions with the transcriptional coactivators CBP and SRC-1 [3]. It has also been demonstrated that glycogen synthase kinase 3 (GSK-3) can mediate phosphorylation of S102, S104, S106, and S118 *in vivo *and *vitro*, where S102 phosphorylation is dependent on pS104 [7]. S167 of ERα is also phosphorylated in response to epidermal growth factor receptor signaling through p90 RSK (p90 ribosomal S6 kinase), thereby significantly enhancing ERα transcriptional activity [8]. This laboratory demonstrated that src kinase dependent activation of AKT resulted in phosphorylation of ERα at S167 and this site was necessary for src mediated ERα transcriptional activity [4]. Additionally, protein kinase CK2 which is upregulated in most proliferating tissues, phosphorylates S167 and regulates interaction of ERα with estrogen response elements (ERE) *in vitro *[9,10].

In addition to phosphorylation sites that have been functionally characterized, recent studies have identified novel phosphorylation events at sites S102, S154, S212, S294, S554, and S559 by mass spectrophotometry [11,12]. Concurrent studies described herein have confirmed S294 and S559 as bona fide ERα phosphorylation sites using phospho-peptide mapping and have ascribed the initial functional significance of these sites to ERα transcriptional activity. Additionally, antibodies utilized within this study have detected *in vivo *phosphorylation of S282, S294, and S559 in immunohistochemical analysis of human breast carcinoma tissue microarrays [13].

Until recently, evidence for a role of ERα phosphorylation in breast cancer had been extrapolated from breast cancer cell line models. However, recent studies demonstrate that ERα phosphorylation may significantly impact ERα signaling in human tissues. Immunohistochemical studies have demonstrated S118 phosphorylation of ERα in breast cancer patient biopsies [6,14]. S118 phosphorylation was associated with improved disease free survival despite the ability of S118 phosphorylation to mediate ligand independent ERα function and association of S118 phosphorylation with EGFR signaling, a known contributor to tamoxifen resistance in tissue culture models [15,16]. Furthermore, S118 phosphorylation was directly associated with tamoxifen sensitivity as well as with a more highly differentiated tumor phenotype [17,18]. Another study, however, found that ERα S118 phosphorylation was related to Her2 expression and tamoxifen resistance upon patient relapse [19]. S167 phosphorylation has also been correlated to responsiveness to endocrine therapy as well as increased disease free and overall survival in breast cancer patients [20]. Interestingly, S167 is downstream of AKT signaling which has been associated with tamoxifen resistance and agonist activity in endometrial cancer cells [4]. Most recently, it has been reported that low levels of S118 phosphorylation accompanied by high levels of S167 phosphorylation were associated with increased overall survival and disease-free survival in a study of breast cancer patient biopsies [21]. Furthermore it was recently suggested that phosphorylation of serine 305 in premenopausal women with breast cancer was related to tamoxifen resistance [22]. Although the number of clinical studies correlating ERα phosphorylation and patient prognosis/outcome are relatively few, these studies present the possibility that ERα phosphorylation could be predictive of responsiveness to endocrine therapy in ERα positive breast cancer.

A number of reports indicate that the most studied of the previously identified ERα phosphorylation sites are not the critical targets of signaling pathways that regulate the relative antagonist or agonist properties of tamoxifen. Three N-terminal serine phosphorylation sites (S104, S106, S118) in ERα were shown to be necessary, but not sufficient for growth factor potentiation of ligand-dependent activation and ligand-independent activation of the ERα [5,23-25]. Of significance from these studies was the recognition that at least three otherERα serine phosphorylation sites remained to be identified *in vivo*.

With the expectation that additional ERα phosphorylation sites may play a primary role in the modulation of ERα function, the present study sought to identify novel ERα phosphorylation sites employing *in vivo *labeling of mammalian cells with [P^32^]H_3_PO_4_, phosphopeptide mapping and biochemical identification of sites. The present study has identified four novel ERα phosphorylation sites *in vivo *at serine residues S46/47, 282, 294, and 559. The identification and characterization of these novel ERα phosphorylation sites will provide further insight into ERα function in both normal and disease states.

## Results

### Identification of serines 47, 282, 294, and 559 as novel ERα phosphorylation sites

**Figure 2:**
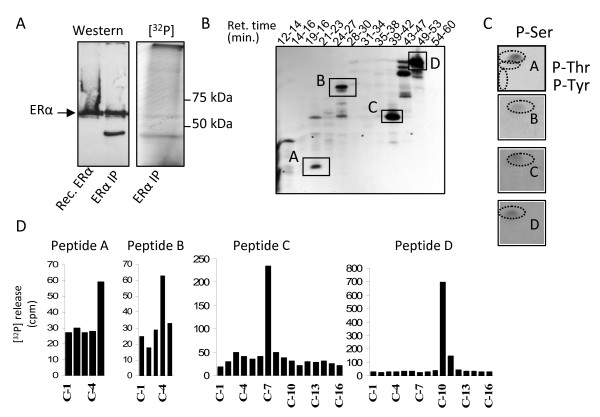
**Identification of ERα phosphorylated at serine residues 47, 282, 294 and 559**. 6 × 10^8 ^COS-1 cells were cultured in phenol red free DMEM supplemented with 10% charcoal stripped FBS. Cells were transfected with wt ERα expression plasmid as noted in materials and methods. 24 hours post-transfection, cells were phosphate-depleted and medium was exchanged with phosphate free DMEM supplemented with 1% dialyzed FBS. 4 mCi [P^32^] H_3_PO_4 _and 10^-8 ^M estradiol were added to each plate and incubated overnight. Cells were subjected to denaturing lysis, ERα purified by immuno-affinity column, eluted, and fractionated by SDS-PAGE. **(A) **The band corresponding to the 67 kDa ERα was excised and subjected to tryptic digestion. Tryptic ERα peptides were separated by reverse phase HPLC using a C-18 column and 0-45% acetonitrile gradient over 90 minutes. **(B) **Fractions were collected, pooled according to HPLC retention times and electrophoresed on 40% acrylamide alkaline peptide gels. Gels were autoradiographed, revealing distinct ERα phosphopeptides. 4 novel phosphopeptides (A, B, C, and D) resulting from tryptic digestion of ERα were identified. Each phosphopeptide was then excised, and subjected to modified manual Edman degradation (MED) and phosphoamino acid analysis as described in Materials and Methods. **(C) **Phosphoamino acid analysis revealed that the phosphopeptide A, B, C and D contained only phosphoserine. Representative phosphoamino acid analyses autoradiograms are presented with identical results detected for phosphopeptides A, B, C, and D. **(D) **MED detected ^32^P release for phosphopeptides A-D. Combined data for phosphoamino acid analysis and MED is presented in Table 1.

To identify hitherto unidentified ERα phosphorylation sites, the present study employed the same approach used by this laboratory to identify phosphorylation sites in coactivator SRC-1 [26]. Briefly, COS-1 cells expressing ERα were labeled *in vivo*, with [P^32^]H_3_PO_4 _and ERα was immunopurified and fractionated by SDS-PAGE. A small aliquot was used to confirm purification of ERα (Figure 2A). Following autoradiography of the wet gel, the 67Kd band corresponding to ERα was excised, subjected to tryptic digestion, and ERα tryptic peptides were separated on a C-18 reverse-phase HPLC column using a 0-45% acetonitrile gradient. Radiolabeled peptides were collected and electrophoresed on a 40% alkaline acrylamide gel and autoradiographed to reveal sites of P^32 ^incorporation corresponding to ERα phosphorylation (Figure 2B). Subsequently, phosphopeptides were subjected to phosphoamino acid analysis and modified manual Edman degradation to determine the position of each phosphoamino acid within the phosphopeptides. Data was obtained for individual phosphopeptides isolated from HPLC fractions or alkaline polyacrylamide gels containing each of the four major phosphopeptides labeled A-D in Figure 2B. Phosphoamino acid analysis indicated that each phosphopeptide contained only phospho-serine (Figure 2C). Modified manual Edman degradation determined the position of P^32^phosphorylated amino acids within each phosphopeptide as follows: phosphopeptide A, P^32 ^release at cycle 5; phosphopeptide B, P^32^release at cycle 4; phosphopeptide C, P^32 ^release at cycle 7, and; phosphopeptide D, P^32 ^release at cycle 10 (Figure 2D). Comparison of these results to the predicted tryptic peptides of ERα revealed a single candidate phosphopeptide for phosphopeptide A and for phosphopeptide B (Table 1). The phosphorylation site in phosphopeptide A was identified as serine 282 and the site in phosphopeptide B was identified as serine 559. S559 phosphorylation has been recently reported by Atsriku *et al*. through MALDI-TOF mass spectrophotometry in ERα + MCF-7 cells, thereby validating that S559 is a phosphorylation site for ERα [11]. For phosphopeptide C there were four candidate peptides predicted to have a cycle 7 serine release by manual Edman degradation (Table 1). S294 was a predicted substrate for Ser/Pro directed kinases and this sequence is conserved among nuclear receptors (see Table 2). Two of the other three candidates for phosphopeptide C were not predicted to be phosphorylation sites according to NetPhosK phosphorylation prediction models [27]. The fourth candidate, S527, was a predicted substrate for PKC but the sequence is not conserved among nuclear receptors. Therefore of the four candidate phosphopeptides, S294 was the most likely candidate for the phosphorylated residue contained within peptide C. As with S559, S294 has recently been confirmed as a ERα phosphorylation site by MALDI-TOF mass spectrophotometry in MCF-7 cells [11]. Similarly for phosphopeptide D, two possible peptides were predicted to have a cycle 10 serine release by manual Edman degradation; either S47 or S193. S47 was contained in an imperfect consensus sequence for PKC whereas S193 was not within a predicted kinase recognition sequence (Table 1).

**Table 1:** Identification of novel ERα phosphorylation sites.

Peptide	Amino acid	MED Cycle	Candidate phosphopeptides	Identity	Verified by mutagenesis	Phospho- antibody
**A**	**Serine**	**5**	***278-****GEVG ****(S) ****AGDMR****-287***	**Serine 282**	**+**	**+**
**B**	**Serine**	**4**	***556-****GGA ****(S) ****VEETDQSHLATAGSTSSHSLQK****-581***	**Serine 559**	**+**	**+**
**C**	**Serine**	**7**	172-GSMAME (**S) **AK-180 ***288-****AANLWP ****(S) ****PLMIK****-299*** 450-SIILLN(**S)**GVYTFLSSTLK**-**467 521-GMEHLY(**S)**MK**-**529	**Serine 294**	**+**	**+**
**D**	**Serine**	**10**	***38-****PLGEVYLDS ****(S) ****KPAVYNYPEGAAYEFNAAAA* *ANAQVYGQTGLPYGPGSEAAAFGSNGLGGFPPLNSV(****S*)***P(****S*)***PLMLLHPPPQL (****S*) ***PFLQPHGQQVPYYLENE* *PSGYTVR****-142*** 184-YCAVCNDY **(S) **GYHYGVWSCEGCK-206	**Serine 47**	**+**	**-**

**Table 2:** Consensus kinase recognition sequences for novel ERα phosphorylation sites

Phosphorylation site	Sequence	Consensus recognition site	Putative Kinase
**Serine 47**	YLD-**(*S*)-*(S)K*-**PAV	**(S/T)**-X-R/K	PKC? (imperfect)
**Serine 282**	GEVG**-*(S)AGD-***M	**(S/T)**-X-X-E/D	CK2
**Serine 294**	NLWP**-*(S)P*-**LMI	**(S/T)**-P	Proline-directed kinase
**Serine 559**	RGGA**-*(S)VEE*-**T	**(S/T)**-X-X-E/D	CK2

**Figure 3:**
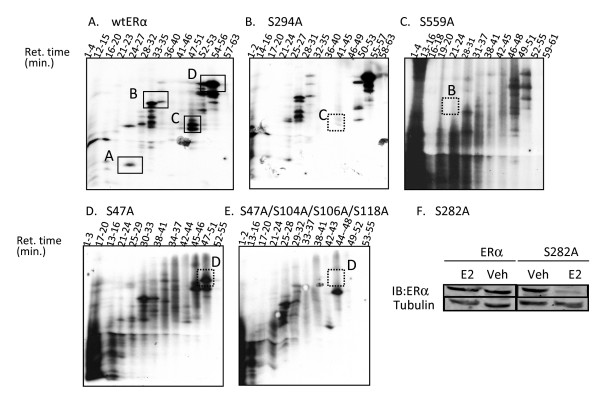
**Mutation of serine residues to alanine eliminates specific phosphorylation of peptides**. To confirm the identity of phosphorylated serine residues within peptides A, B, C, and D, serine to alanine mutations were introduced into wt ERα (S47A, S282A, S294A, or S559A). 12 plates of COS-1 cells (4 × 10^7 ^/plate) were transfected with 500 ng/plate of wt ERα, S47A, S282A, S294A, or S559A expression plasmids. 18 hours post-transfection, cells were phosphate-depleted, labeled with 4 mCi [^32^P]H_3_PO_4 _and incubated with 10^-8 ^M estradiol overnight. ERα was immunopurified and tryptic peptides were separated by HPLC using a C-18 reversed phase column. Fractions were collected and electrophoresed on a 40% alkaline polyacrylamide gel followed by autoradiography. **(A) **Peptide map of wt ERα displaying 4 novel phosphopeptides A-D. **(B) **S294A resulted in loss of peptide C. **C) **S559A resulted in loss of peptide B. **(D) **S47A resulted in a modest decrease in peptide D compared to wt ERα. **E) **S47A/S104A/S106A/S118A resulted in loss of peptide D. **(F) Mutation of S282 to alanine reduces ERα protein following 24 h incubation with estradiol**. 10^6 ^COS-1 monkey embryonic kidney cells which had been cultured in phenol-red free DMEM supplemented with 10% fetal bovine serum were transfected with 2.5 μg of wt ERα or S282A expression plasmid. 24 hours after transfection, cells were incubated with vehicle (veh) or estradiol (10^-8^M) for an additional 24 hours. Cell lysates were collected and ERα protein levels determined, using α-tubulin as a loading control.

To distinguish among the candidate peptides for phosphopeptides C and D, and to provide further confirmation of the identity of all phosphorylation sites in phosphopeptides A-D, serine to alanine mutant ERα expression constructs were generated at sites S47, S282, S294, and S559 (S47A, S282A, S294A, S559A). ERα mutants were expressed in COS-1 cells and P^32^phosphopeptide maps were prepared and compared to phosphopeptide maps of wild-type (wt) ERα (Figure 3A-E). Phosphopeptide maps of S294A or S559A resulted in specific loss of phosphopeptides C and B, respectively (Figure 3B-C). The phosphopeptide map of S47A resulted in reduced intensity but not complete loss of phosphopeptide D (Figure 3D). A close examination of the first tryptic cleavage site directly C-terminal to S47 revealed a lysine-proline sequence at residues 48-49. Trypsin cleaves inefficiently at R/K-proline sequences making it possible that phosphopeptide D was the result of an incomplete tryptic digest [28]. The next trypsin cleavage site following an incomplete digest at K48 occurs at residue R142 and digestion at this site would result in a very large phosphopeptide of 110 amino acids. The possibility of an incomplete tryptic digest at K48 was consistent with the relative migration of phosphopeptide D at the top of the alkaline acrylamide gel, and the elution of this phosphopeptide in the later fractions from the C18 reversed phase column (25). Since the previously identified ERα phosphorylation sites S104, S106, and S118 would also be present in the very large phosphopeptide resulting from incomplete digestion of K48, an ERα expression plasmid was constructed containing four serine-to-alanine mutations at S47, S104, S106, and S118 and a phosphopeptide map of this protein was prepared. As shown in Figure 3E, S47A/S104A/S106A/S118A resulted in complete loss of phosphopeptide D consistent with the interpretation that all four phosphorylation sites S47, S104, S106, and S118 were present in phosphopeptide D. Additionally, it was noted that phosphopeptide C was diminished upon mutation of S104, S106, S47 and S118, suggesting that S294 phosphorylation, or a site within a peptide which co-migrates electrophoretically with peptide C may be dependent upon phosphorylation of one or several sites within the D peptide (Figure 3D and 3E).

Despite multiple attempts, phosphopeptide maps of S282A consistently resulted in a very weak steady state ERα phosphorylation profile. This was likely the result of estradiol induced loss of S282A protein after 24 hours (Figure 3F). This effect was time dependent since estradiol did not reduce S282A protein after 3 hours incubation. The destabilization of ERα by mutation of S282A is currently under investigation. The effect of phosphorylation site mutation on phosphorylation at other sites is also under investigation.

### Phospho-specific antibodies recognize ERα phosphorylated at serines 282, 294, and 559

**Figure 4:**
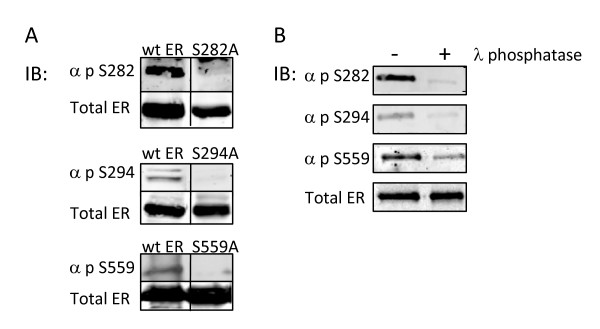
**Confirmation of the specificity of ER phospho-antibodies**. **(A) **Serine to alanine mutations at ERα phosphorylation sites inhibit reactivity of phospho-specific antibodies. COS-1 cells cultured in DMEM growth supplemented with 10% FBS were transiently transfected with 500 ng of either wt ERα or serine to alanine substituted ERα expression plasmids (S47A, S282A, S294A, or S559A). 18 hours post-transfection, cells were lysed and subjected to Western immunoblot analysis utilizing custom polyclonal antibodies directed toward the individual phosphorylated ERα residues (S47, S282, S294, or S559) or monoclonal ERα antibody as indicated. α-p-S282, α-p-S294, or α-p-S559 antibodies did not recognize S282A, S294A, or S559A, respectively, indicating phospho-antibody specificity. Mutation of S47 failed to eliminate immunoreactivity of αp-S47. **(B) **In vitro λ phosphatase treatment of ERα inhibits immunoreactivity of ERα phospho-antibodies. Baculovirus expressed ERα was subjected to dephosphorylation by λ phosphatase for 30 minutes at 30°C and analyzed by Western immunoblot with antibodies against p-S282, p-S294, p-S559, and total ERα. Dephosphorylation inhibited immunoreactivity of, α-p-S282, α-p-S294, and α-p-S559 without impacting immunoreactivity of total ERα antibody.

**Figure 5:**
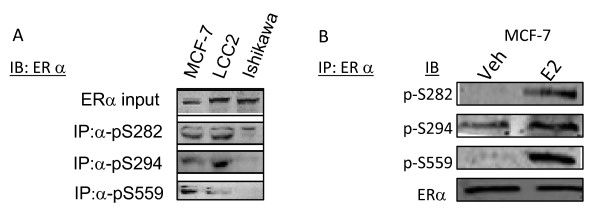
**Phosphorylation of endogenous ERα in ERα (+) cell lines at S282, S294, and S559**. (**A) **10^7 ^MCF-7 and MCF-7-(LCC2) breast cancer and Ishikawa endometrial adenocarcinoma cell lines were cultured in medium supplemented with 10% FBS. Cells were lysed and total ERα was immunoprecipitated with α-p-S282, α-p-S294, or α-p-S559 antibodies for 3 hours. Immunoprecipitates were analyzed by Western blot for total ERα (**B) **S282 and S559 are phosphorylated following incubation of MCF-7 breast cancer cells with estradiol. MCF-7 breast cancer cells were cultured for 48 hours in phenol red free medium supplemented with 10% charcoal-stripped FBS. Cells were serum starved overnight prior to incubation with vehicle (veh) or 10^-8^M estradiol (E2). ERα was immunoprecipitated from lysates and Western blot analysis performed with α-pS282, α-pS294, or α-pS559, and with α-ERα. Substantial ligand-induced phosphorylation was detected at S282 and S559, with only modest ligand induced phosphorylation at S294.

To further verify the authenticity of the phosphorylation sites identified by the biochemical approaches above and to study the impact of ERα phosphorylation at S47, S282, S294, and S559, phospho-specific antibodies to each site were generated. Immunogens (phosphorylated peptides) were designed and rabbit polyclonal antibodies were generated against each phosphorylation site as previously described by this laboratory [29]. To validate the phospho-specific antibodies, S47A, S282A, S294A or S559A were expressed in COS-1 cells and lysates were subjected to Western immunoblotting with total ERα antibody and with phospho-specific antibodies to each site (Figure 4A). Each point mutation was expressed at comparable levels to wt ERα. Each phospho-specific antibody recognized wt ERα but the phospho-specific antibodies to sites S282, S294 and S559 did not recognize the corresponding mutations S282A, S294A, and S559A confirming that phosphorylation at these sites was necessary for the immuno-reactivity. In contrast, mutation S47A was still recognized by the phospho-antibody to pS47. Of interest was the decreased electrophoretic mobility of ERα upon mutation of S47 to alanine (Figure 5C). This reduced mobility may be indicative of further post-translational modifications that are regulated by S47 phosphorylation similar to what has been observed for S305A mutations (27).

To further validate the specificity of the ERα phospho-specific antibodies, baculovirus expressed recombinant ERα was incubated with and without λ-phosphatase followed by Western blotting with phospho-specific antibodies. Each antibody was determined to be phosphorylation state specific as evidenced by the lack of immunoreactivity following incubation with λ-phosphatase (Figure 4B).

The results for α-pS47 were inconclusive since mutation of this residue failed to inhibit immunoreactivity, yet dephosphorylation by λ phosphatase indeed decreased immunoreactivity (data not shown). These findings lend evidence to the existence of an alternative phosphorylation site within the region corresponding to the immunogenic peptide used to generate the pS47 antibody. It was therefore investigated whether phosphorylation of the adjacent serine 46 (S46) was responsible for the immunoreactivity of the α-p-S47 antibody. Results indicate that mutation to alanine of either S46, S47 or S46/S47 did not block immunoreactivity of the p-S47 antibody to ERα (data not shown) indicating that the α-p-S47 antibody was not specific for the Western blotting procedure. As such, the α-pS47 antibody was not used for subsequent studies. It is possible this antibody may demonstrate specificity in other procedures (e.g. IHC) and this is currently being investigated.

### Endogenously expressed ERα is phosphorylated at S282, S294, and S559

To determine whether endogenously expressed ERα was phosphorylated at sites S282, S294, and S559, ERα was first immunoprecipitated using phosphospecific antibodies directed towards p-S282, p-S294, and p-S559, followed by Western blotting with antibody to total ERα in a panel of ERα+ breast cancer cell lines. Using this approach, phosphorylation at each site was detected in MCF-7 breast cancer cells and MCF-7-LCC2 cells, a tamoxifen resistant derivative of MCF-7 [30]. ERα+ Ishikawa endometrial adenocarcinoma cells did not exhibit substantial phosphorylation at S559, and weak immunoreactivity for S294 and S282 phosphorylation observed. These studies indicate that ERα is phosphorylated at serines S282, S294, and S559 in endogenous ERα expressing breast and endometrial cancer cells. It should be noted that phosphospecific antibodies for p-S282, p-S294, and p-S559 also detected phosphorylated ERα in ERα positive human breast carcinomas [13].

**Figure 6:**
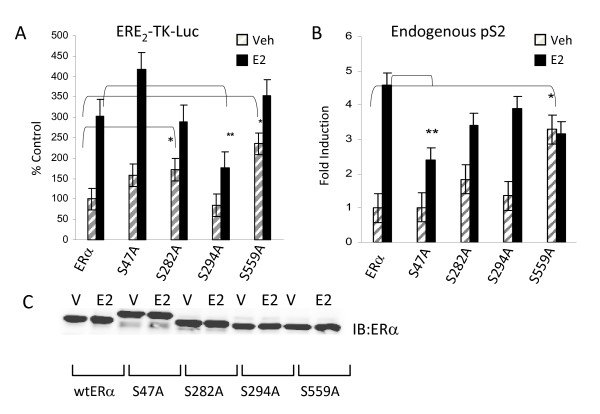
**Phosphorylation of ERα impacts receptor transcriptional activity**. **(A) **ERα (-) HeLa cells were cotransfected with 100 ng ERE_2_-TK-luciferase reporter and 200 ng wt ERα (wt), or serine to alanine mutants of ERα for each novel phosphorylation site (S47A, S282A, S294A, and S559A). 24 hours post transfection, cells were incubated with vehicle (veh) or estradiol (10^-8 ^M) overnight. Luciferase assays were performed and transcriptional activity was normalized to protein concentration and/or ERα expression by Western blot analysis. S47A exhibited similar transcriptional activity to wt ERα, whereas S294A resulted in suppressed transcriptional activity vehicle and estradiol. S282A and S559A displayed enhanced ligand independent transcriptional activity as compared to wt ERα. **(B) **HeLa cervical cancer cells were transfected with 500 ng of wt ERα or ERα phospho-mutant (S47A, S282A, S294A, S559A) expression plasmids. 24 hours post-transfection, cells were incubated with vehicle (veh), 10^-8 ^estradiol (E2), for 3 hours. pS2 expression was measured by real-time RT-PCR, relative to GAPDH. S47A resulted in suppression of estradiol-induced pS2 expression, whereas S559A exhibited ligand-independent activation of ER. S282A and S294A displayed no statistical differences in pS2 mRNA. **(C) **HeLa cells were transfected with 500 ng of wt ERα or ERα phospho-mutant (S47A, S282A, S294A, and S559A) expression plasmids and incubated for 3 hours with estradiol (1^-8^M) at 18-24 hours post transfection. Transfection and incubation with estradiol were performed in parallel with those for RT-PCR (panel B). A and B represent composite results for 6 identical experiments. Statistical significance was determined using ANOVA and Fisher's LSD post-hoc analysis, p ≤ 0.05.

To determine if the phosphorylation of ER at S282, S294, or S559 was regulated by estradiol, MCF7 cells were incubated for 30 minutes with 10^-8 ^M estradiol. Estradiol markedly increased phosphorylation at sites S282 and S559 but had little effect on S294 (Figure 6B). These data further demonstrate that phosphorylation of ERα at S282, S294 and S559 comprise an integral component of endogenous ERα signaling.

### ERα phosphorylation at novel sites impacts ERα regulated gene expression

To determine the role of ERα phosphorylation in receptor mediated transcription, ERα negative [ERα(-)] HeLa cervical cancer cells were cotransfected with individual ERα phospho mutant expression plasmids and a reporter plasmid (ERE_2_-TK-luciferase) containing two canonical ERE sequences. Mutation of a serine residue to non-phosphorylatable alanine mimics loss of a phosphorylation site. S282A and S559A exhibited a significant increase in basal reporter activity compared to wt ERα but these mutants exhibited no statistical difference compared to wt ERα in estradiol-dependent reporter activation (Figure 5A). S294A exhibited reduced estradiol-dependent reporter activation. S47A mutants showed a trend toward increased basal and ligand dependent activation in reporter assays, but did not reach statistical significance. These data suggest that phosphorylation at sites S282 and S559 inhibit ligand-independent activation of ERα, and that phosphorylation at S294 is required for full estradiol activation of gene transcription.

Real time RT-PCR was used to measure expression of the endogenous estrogen responsive gene pS2 in HeLa cells transiently transfected with wt ERα or mutant ERα expression plasmids. The pS2 promoter contains separate ERE and AP-1 sites [31,32]. Most intriguing and consistent with the reporter gene results in Figure 5A, expression of S559A resulted in significantly elevated ligand independent activation of pS2 (Figure 5B). Similarly, S282A also resulted in elevated ligand independent activation of pS2. S47A exhibited significant repression of estradiol-induced pS2 expression, which was in contrast to what was observed with the reporter gene assay. S294A exhibited no change in either basal or estradiol induced pS2 expression compared to wt ERα. The differential effects observed with regard to S47A and S294A regulation of pS2 versus the ERE_2_-TK-luciferase reporter gene likely reflect gene specific effects for ERα phosphorylation. Interestingly, the ERE of the pS2 gene works in synergy with an adjacent AP-1 site to mediate estradiol induced pS2 expression [31,33]. Figure 5C demonstrates that an equivalent level of ERα protein was detected for wt ERα and the mutants.

One limitation of ERα(-) cells such as HeLa to study pS2 or other endogenous gene expression, is that these cells do not express pS2 and cells transiently transfected with ERα expression plasmids likely produce low levels of pS2 protein following estradiol treatment due to limitations of the transient transfection approach. Future studies will directly mutate ERα phosphorylation sites of the endogenous ESR-1 gene in cells that robustly express ERα to overcome the limitations of transient transfection employed here.

**Figure 7:**
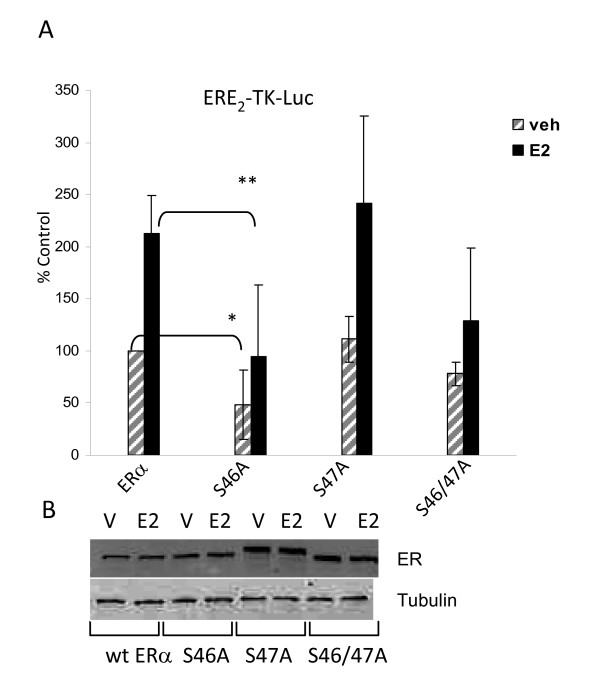
**Mutation of S46 and S46/47 impacts receptor transcriptional activity**. **(A) **10^5 ^ERα (-) HeLa cells were cotransfected with 100 ng ERE_2_-TK-luciferase reporter and 200 ng wt ERα (wt), or serine to alanine mutants of ERα for S46 (S46A), S47A (S47A), or both S46 and S47 (S46/47A). 24 hours post transfection, cells were incubated with vehicle (veh) or estradiol (10^-8 ^M) for 18-24 hours. Luciferase assays were performed to determine the relative transcriptional activity of ERα and ERα phospho-mutants. Transcriptional activity was normalized to protein concentration and ERα expression by Western blot analysis. These studies demonstrate that S46A and S46/47A lead to substantial inhibition of ERα mediated gene expression, whereas the activity of S47A remains similar to that of wt ERα. **(B) **Western blot analysis demonstrating expression of wt ERα, S46A, S47A, and S46/47A. 10^5 ^HeLa cells were transfected with 500 ng of wt ERα or ERα phospho-mutant (S46A, S47A, or S46/47A) expression plasmids and incubated for 5 hours with estradiol (1^-8^M) at 18-24 hours post transfection. S47A shows an electrophoretic upshift not evident with wt ERα, S46A, or S46/47A. Panel A represents the composite of 3 experiments. Statistical significance was determined using ANOVA and Fisher's LSD post-hoc analysis, p ≤ 0.05.

Although biochemical identification and site directed mutagenesis identified S47 as a novel phosphorylation site in phosphopeptide D, the α-pS47 phosphospecific antibody retained immunoreactivity with S47A. Since λ phosphatase resulted in loss of immunoreactivity of α-pS47 antibody the possibility was raised that the adjacent serine, S46, might be phosphorylated in the S47A mutant. NetPhosK software revealed that S46 forms a putative recognition sequence for protein kinase C classic isoforms that more closely resembles a canonical recognition motif for PKC classical isoforms than does S47. To investigate the possibility that S46 phosphorylation could be an alternative phosphorylation site for S47 of ERα, serine to alanine mutations were introduced at S46 and/or S47 (S46A, S46/S47A). ERα(-) HeLa cervical cancer cells were cotransfected with S46A, S47A, or S46/47A expression plasmids and ERE_2_-TK-luciferase reporter plasmid. Results indicated that mutation of S46, but not S47, significantly suppressed ERα transcriptional function (Figure 7A). The 46/47A mutant displayed similar activity to S46A, suggesting that the predominant effect on transcriptional activity was through S46 phosphorylation.

### Protein kinase CK2 phosphorylates ERα S282 and S559 *in vitro *and *in vivo*

**Figure 8:**
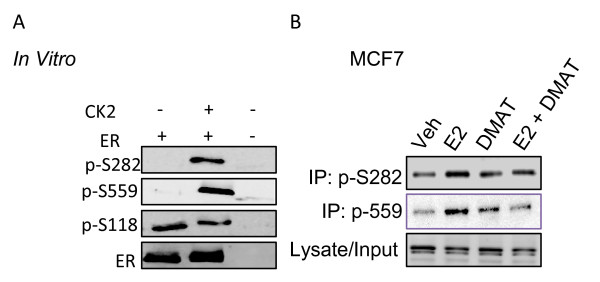
**Protein Kinase CK2 phosphorylates of S282 and S559**. **(A) **400 ng baculovirus expressed ERα was incubated in CK2 kinase buffer supplemented with 10 mM ATP, in the presence or absence of 200 ng recombinant catalytic α subunit of CK2. Reactions were stopped with Laemmli buffer, subjected to Western blot analysis, and probed with α-pS282, α-pS559, α-pS118 or αER. These studies show that the CK2α catalytic subunit specifically phosphorylates ERα at S282 and S559. Western blot for phosphorylation of S118, a site that exhibits strong phosphorylation in baculovirus expressed ERα, is shown for comparison to demonstrate absence of nonspecific phosphorylation by CK2 on other ERα phosphorylation sites. **(B) **10^6 ^MCF7 breast cancer cells were pretreated with DMAT (4 uM) for 90 minutes, followed by 30 minutes with estradiol (10^-8 ^M) or vehicle. Immunoprecipitation of S282 or S559 was performed using phosphoantibodies and Western blot for total ERα. DMAT inhibited phosphorylation at both sites, indicating that CK2 phosphorylates these sites *in vivo*.

Motif analysis revealed that each phosphorylation site was contained within known kinase recognition motifs (Table 2). S46/47 was present within an imperfect recognition motif for classical PKC isoforms (S/T-X-K/R). S282 and S559 were present within a consensus sequence for protein kinase CK2 phosphorylation (S/T-X-X-E). S294 was present within a putative recognition motif for Ser/Pro directed kinases. *In vitro *phosphorylation of baculovirus expressed human ERα was performed with ERK1/2 (to assess S294 phosphorylation), and protein kinase CK2α catalytic subunit (to assess S282 and S559 phosphorylation) followed by Western blotting with phospho-specific antibodies. *In vitro *kinase assays with ERK1/2 did not result in phosphorylation at S294 (data not shown). Incubation of ERα with protein kinase CK2 resulted in phosphorylation of S282 and S559 *in vitro *(Figure 8A). These findings are of significant interest with regard to CK2 overexpression documented in various cancers, including breast cancer [34].

To demonstrate the involvement of CK2 in the phosphorylation of ERα at S282 and S559 *in vivo*, MCF7 cells were incubated with the highly selective CK2 inhibitor, DMAT (2-dimethylamino-4,5,6,7- tetrabromo-1H-benzimidazole) for 1 hour, prior to incubation with E2 for 30 minutes. Cell lysates were subjected to immunoprecipitation with α-pS282 and α-pS559 antibodies, and subsequently Western blots using antibody to total ERα protein. DMAT suppressed phosphorylation of S282 and S559 further indicating that CK2 is responsible for the phosphorylation of both S282 and S559 of ERα (Figure 8B).

## Discussion

The present study has characterized four novel phosphorylation sites in human ERα *in vivo*. Two of these sites, S294 and S559, have been independently confirmed by mass spectrophotometry in MCF-7 cells [11]. Additionally, collaborators at the University of Manitoba recently found that ERα is phosphorylated at S282, S294, and S559 in human breast carcinoma biopsies, lending evidence to the physiological relevance of these sites in the breast cancer [13]. S559 phosphorylation is the first phosphorylation site identified in the extreme C-terminal (F domain) of a steroid nuclear receptor [35]. Generation of phospho-specific ERα antibodies provided the tools to validate phosphorylation sites and to begin to assess the functional significance of these specific sites. Accordingly, these phospho-specific antibodies were used to detect phosphorylation of each residue by Western blot/immunoprecipitation from cells that expressed endogenous ERα (Figure 6A and 6B). Phosphorylation at S46/47, S282, S294 and S559 each had an impact on ERα mediated activation of reporter gene ERE_2_-TK-luciferase and/or endogenous pS2 gene expression (Figure 5A and 5B). Two of these sites, S282 and S559, were contained within consensus sequences for protein kinase CK2 and were phosphorylated by CK2 *in vitro *and *in vivo *(Figure 7, 8). In particular, phosphorylation changes at the F domain S559 site had profound effects on ligand independent activation of ERα. These findings provide valuable insight into the regulation of ligand dependent and ligand independent ERα function. Furthermore, pharmacological targeting of the cell signaling pathways that regulate ERα phosphorylation at these sites may represent therapeutic targets for modulating ERα function.

S559, located in the F-domain of ERα, represents a very intriguing phosphorylation site because mutation to alanine enhances ligand independent activation of ERα (Figure 5A and 5B). Studies have demonstrated that the F-domain of various nuclear receptors may be responsible for interaction with coregulators, and for determining the relative agonist/antagonist character of several ERα ligands [36]. At least two groups have demonstrated that F-domain deletions, that included deletion of a regions containing S559, enhanced transcriptional activity of ERα [37,38]. Furthermore, SRC-1 association with ERα was increased upon deletion of the F domain [39]. Another report demonstrated that the intact ERα F-domain inhibited the association of unliganded ER with REA (Repressor of Estrogen Receptor) using *in vitro *GST pull down assays [40]. These studies indicate that the F-domain of ERα was responsible for the inhibition of ERα association with both coactivators (SRC-1) and corepressors (REA). Although these findings do not directly implicate a central role for S559 phosphorylation in coregulator recruitment, it does highlight the importance of the F-domain in ERα/coregulator interactions. Interestingly, another report suggested that point mutations of S559 and E562 to alanine disrupted a predicted helix in the F-domain that resulted in a slight inhibition of ligand independent transcriptional activity and an enhanced estradiol-activation of ERα mediated gene expression [38].

The phosphorylation of S282 and S294 may also be important for regulating ERα function. The location of S282 and S294 in the hinge region near the DBD is suggestive of a role for these phosphorylation sites in DNA binding and/or dimerization of ERα. The present data, however suggest that phosphorylation of these residues lead to opposing effects on ERα mediated transcription (Figure 5A and 5B). Reporter gene data suggest that phosphorylation at S282, much like S559, was involved in the suppression of unliganded ERα activity. However, mutation of S282 also resulted in increased estradiol-dependent loss of ER protein. As such, the impact of S282 phosphorylation may be biphasic. In the transient setting, S282 phosphorylation may result in attenuated transcriptional activity but may lend to long term stabilization of ER protein levels allowing transcriptional activity to persist over time. S294 mutation to alanine resulted in a suppressive effect in reporter assays but not in expression of the endogenous estrogen regulated gene pS2. One likely explanation is that the complexity of the endogenous pS2 promoter allows other transcription factors/transcriptional regulators to compensate for loss of ERα function caused by mutation of S294 to alanine. The pS2 promoter does indeed contain an AP-1 site that is thought to act in concert with the ERE to mediate transcriptional activity. In this setting, slight changes in ERE mediated transcriptional function may be compensated with the AP-1 site.

S46/47 phosphorylation may play a role in the ligand dependent activation of ERα. Data suggested that S46, and not S47, is responsible for regulating ERα transcriptional activity. S47 which was identified by biochemical means, may be phosphorylated due to a "bystander effect" in which kinases, (presumably PKC isoforms) are directed to the S46 site but may phosphorylate both the S46 and the weaker recognition site, S47. Alternatively, S46 and S47 phosphorylation events could be distinct events. Indeed, S46 lies within an imperfect protein kinase C recognition motif as does S47 and mutation of S46 results in substantial inhibition of ERE-luciferase activity in HeLa cells but was not identified biochemically as an ERα phosphorylation site. Interestingly, mutation of S46 or S46/47 does not lead to the electrophoretic up shift observed upon mutation of S47 alone suggesting that these sites play distinct roles in ER transcriptional regulation. These data can only be suggestive of S46 phosphorylation as there has been no direct biochemical evidence that S46 is phosphorylated. As with the other four A/B region phosphorylation sites (S104, S106, S118, S167), S47 may contribute to the ligand independent and ligand dependent function of the receptor. In agreement with this assertion it has been demonstrated that murine ERα transcriptional activity is induced by PKCδ through the AF-1 domain and that PKCδ activity leads to phosphorylation of mouse ERα [41]. Nonspecificity of the α-pS47 antibody precluded further experiments that would have been necessary to determine if PKC is indeed necessary for the phosphorylation of S46/47 and if this mediates the transcriptional induction of ERα through the PKC pathway.

The finding that S282 and S559 were phosphorylated *in vivo *and *in vitro *by protein kinase CK2 indicates a central role for CK2 in the regulation of ERα transcriptional activity (Figure 7). Previous studies indicate that CK2 enhances ERα DNA binding *in vitro *and that CK2 phosphorylates ERα at serine 167 [9,10,42]. Previous work from this laboratory demonstrated that mutation of S167 to alanine markedly reduced ERα transcriptional activity and disrupted ERα interaction with endogenous promoters [4]. S282 and S559 represent the second and third sites of ERα regulation by CK2. Remarkably, mutation of S282 or S559 to alanine resulted in near opposite functional effects on ERα as compared to mutation of S167 to alanine. ERα ligand independent transcriptional activity was markedly enhanced upon mutation of S282 and S559 to alanine whereas estradiol mediated activation remained equivalent to wt ERα (Figure 5A and 5B). These findings suggested that CK2 phosphorylation maintains ERα in a state in which basal ligand independent activity is suppressed while responsiveness to ligand is retained. This is significant in that CK2 overexpression has been observed in several cancers including breast cancer [34,43]. It has also recently been demonstrated that several tamoxifen resistant breast cancer cell lines showed greater susceptibility to apoptosis upon inhibition of CK2 compared to tamoxifen sensitive MCF-7 cells [44]. Additionally, developmental mouse models reveal a causative role for CK2 in breast cancer in which MMTV-CK2α transgenic mice form mammary tumors at a frequency of 35% as compared to > 1% in wt FVB mice [43]. Therefore, the signaling axis of ERα and CK2 in breast cancer is of great interest and provides a putative target for therapeutic intervention.

The signaling axis between CK2 activity and ERα transcriptional regulation has yet to be elucidated. Though mutagenesis of sites demonstrated that absence of a phosphorylation event at S282 and S559 positively regulated ER transcriptional activity, inhibition of CK2 by DMAT inhibited ER transcriptional activity (data not shown). One possibility to explain these findings is that CK2 may be necessary for the activity of transcriptional coregulators involved in ER signaling. A second explanation may be that ER is phosphorylated by CK2 at additional sites (e.g. S167).

Sequence alignment analysis (Megalign™, DNA*Star) was used to determine conservation of phosphorylation at S47, S282, S294, and S559 among other nuclear receptors. S47, which lies in the highly heterologous and heavily phosphorylated AF-1 region of ERα, was difficult to align with AF-1 regions of other nuclear receptors and consequently it was difficult to assess conservation of this site among receptors. However because there is more homology within the DBD, hinge and LBD regions of nuclear receptors, phosphorylation at sites equivalent to S282, S294 and S559 may be more highly conserved among nuclear receptors. The hAR (human androgen receptor) is phosphorylated by stress kinases (ie. Jun N-terminal Kinase, p38 MAP kinase) within the hAR hinge region (S650), a site that is homologous to and aligns closely with S282 of ERα [45]. Interestingly, the (**S)**PTEE sequence encompassing S650 comprises a putative CK2 recognition motif as does S282, suggesting some conservation in phosphorylation between ERα and hAR. Functionally, S650 phosphorylation of the hAR is suggested to regulate nuclear export [46]. The hPR-B (human progesterone receptor B) is phosphorylated in the hinge region by CDK2 at S676 [47]. However S676 phosphorylation has not been ascribed mechanistic relevance in hPR-B function and this site does not align closely with ERα S282 or hAR S650 in alignment analysis. It is possible however, that phosphorylation of hPR-B at S676 is homologous to ERα S294. S294 is likely targeted by Serine-Proline (Ser-Pro) directed kinases and is located within the hinge domain of ERα. Regarding S559 phosphorylation, to date no phosphorylation site has been identified in the F domain of steroid receptors [35].

Motif analysis identified kinase recognition sequences in other nuclear receptors that may be analogous to CK2 phosphorylation of S282 and S559. ERβ has putative CK2 recognition sites at S234 (**S**ADE) and S513 (**S**PAE). S225 of RXRα (retinoid × receptor alpha) is contained within a putative CK2 site (SANE) that aligns with, and may be homologous to, S282 of ERα. The relatively high number of putative CK2 phosphorylation sites within nuclear receptors may indicate that CK2 phosphorylation is intrinsically linked to cell cycle progression given that CK2 expression and activity is increased in highly proliferating tissues [48].

These studies represent the first characterization of four newly identified phosphorylation sites within ERα. One caveat to these findings is the use of ERα (-) HeLa cells to determine the impact of each phosphorylation site on ERα transcriptional activity. HeLa cells only weakly support AF-1 activity of ERα. As such, these studies may represent mechanisms that are primarily representative of AF-2 regulation. Indeed, three of the four identified phosphorylation sites lie outside of the AF1 domain suggesting that these sites may function independently of AF-1. However this does not preclude phosphorylation dependent interaction between AF-1 and AF-2 domains. Despite these limitations, Hela cells support ERα transcriptional activity in luciferase reporters and demonstrate the regulation of endogenous estradiol regulated genes. Additionally, because the cells are ERα (-), the influence of endogenous ERα expression on estradiol-mediated gene transcription is not present.

## Conclusion

The study presented herein describes the initial identification and/or functional characterization of S46/47, S282, S294, and S559 as phosphorylation sites within ERα. These studies show that phosphorylation at S46/47 or at S294 likely potentiate ERα transcriptional activity whereas S282 and S559 phosphorylation are likely to negatively regulate ERα transcriptional activity as evidence by mutational analysis. Additionally, this study identified protein kinase CK2 as the kinase responsible for the phosphorylation of ERα at S282 and S559. The identification and characterization of phosphorylation at S46/47, S282, S294, and S559 provides additional insight into regulation of ERα signaling. Ultimately, the impact of ERα phosphorylation at these sites will provide new diagnostic tools in breast cancer and may lead to novel therapeutic strategies to target ERα signaling.

## Methods

### Tissue culture and transfection

COS-1 monkey embryonic kidney cells, HeLa human cervical cancer cells, MCF-7 cells (ATCC, Manassas, VA), and MCF-7-LCC2 (from Robert Clarke, Ph.D., Georgetown Univ.) were cultured in Dulbecco's minimum essential medium (DMEM) supplemented with 10% fetal bovine serum (FBS), and 5% penicillin/streptomycin at 37°C and 5% CO_2_. Ishikawa cells (provided by Steven Safe, Ph.D., College Station, TX) were cultured at 37°C with 5% CO_2 _in DMEM supplemented with 5% FBS and 1% penicillin-streptomycin. Prior to incubation with estradiol (10^-8^M) or tamoxifen (10^-7^M), cells were cultured in phenol red-free DMEM or RPMI 1640 supplemented with 10% charcoal dextran stripped FBS, and 5% penicillin/streptomycin for a minimum of 24 hours. Transient transfections were performed using Fugene 6 and in accordance with the manufacturer instructions. To attain higher transfection efficiency, modified inactive (dead) adenovirus was used as a transfection reagent for the *in vivo *labeling experiments as previously described [26]. Briefly, 500 ng plasmid DNA and attenuated adenovirus (multiplicity of infection, 400) were diluted in HEPES buffered saline, to which poly-L-lysine was added to a final concentration of 12.5 ng/ml. Adenovirus/lysine solution was applied to COS-1 cells (2 × 10^6^) plated in serum free media. Two hours post-transfection, DMEM containing 10% charcoal stripped FBS was added to cells.

### *In vivo *labeling and isolation of ERα

This laboratory previously described a detailed procedure for *in vivo *[^32^P]H_3_PO_4 _labeling of nuclear receptors and coactivators in mammalian cells [26,28,49]. Briefly, COS-1 cells were cultured in 150 mm tissue culture plates (4 × 10^7 ^cells/plate) in DMEM supplemented with charcoal dextran stripped FBS. Medium was replaced with serum-free medium and cells were transfected using an adenovirus mediated transfection protocol (see Tissue Culture). Prior to *in vivo *phosphate labeling, transfected cells were incubated for 1.5 hours in serum free, phosphate-free DMEM with 1% penicillin/streptomycin to deplete intracellular phosphate pools. Subsequently medium was replaced with DMEM supplemented with 1% dialyzed FBS (to remove low molecular weight molecules such as phosphates). Immediately after medium exchange, 4 mCi/plate of [^32^P]H_3_PO_4 _along with estradiol (final concentration of 10^-8^M) was added to each plate and cells were incubated at 37°C and 5% CO_2 _for approximately 16 hours overnight. Following removal of medium and several washes with PBS, cells were harvested by scraping, cells were pelleted by centrifugation (500 × g), and protein was extracted using 4 pellet volumes (approximately 2 mls) of denaturing lysis buffer (10 mM Tris pH 8, 50 mM potassium phosphate, 50 mM sodium fluoride, 1 mM sodium vanadate, 2 mM EDTA, 2 mM EGTA, 0.4 M sodium chloride, 5 mM α-monothioglycerol, 8 M urea, and 1 × Pierce HALT protease inhibitor cocktail). Chromatin/DNA in the lysate was sheared by 8-10 passes through a 25 gauge needle and syringe. DNA and cellular debris was removed by ultracentrifugation at 100,000 g for 20 minutes at 4°C. 100 μl of cleared lysate was retained for Western blot analysis while the remaining 2 ml of cleared lysate was diluted 1:20 in PBS/0.1%BSA to dilute the urea and prepare the lysate (total volume 40 ml) for immuno-affinity columns. ERα was purified using an immuno-affinity column consisting of protein A conjugated sepharose beads (500 ml) bound by 0.5 mg mouse monoclonal anti ERα Ab (D12, Santa Cruz). Lysates were passed over columns two times using a peristaltic pump over a period of approximately 2-3 hours. Columns were washed using 300 ml PBS/0.1%BSA/.01% TWEEN 20 over a period of approximately 1 hour. Sepharose beads were collected in PBS, ERα was eluted in 2× Laemmli buffer and the samples were electrophoresed on a 10% SDS PAGE gel. Following autoradiography of the wet gel and comparison to Western blot for ERα, the corresponding ERα band was excised from the wet gel in preparation for trypsin digestion (see below).

### Phosphopeptide mapping

Phosphopeptide mapping of nuclear receptors and coregulators has been previously described [26,28,28,50,51]. Briefly, gel slices corresponding to ERα or ERα phospho-mutants were excised from wet gels and incubated for 30 minutes in 50% methanol/HPLC H_2_0 followed by brief washes in HPLC-grade water to remove SDS and electrophoretic salts from the sample. Gel slices were then digested with 10 μg trypsin in 50 mM ammonium bicarbonate buffer for 4 hours. 10 μg of additional trypsin was added in 3 hour intervals for a total addition of 40 μg trypsin. Samples were lyophilized overnight in a SpeedVac, and the samples were resuspended in 500 μl 50% formic acid solution in preparation for HPLC separation. The sample containing tryptic ERα peptides was loaded onto a C-18 reversed phase column (Vydac) using a Beckman Coulter System Gold^® ^HPLC. A 0-45% acetonitrile gradient over 90 minutes was used to elute phosphopeptides from the column. Each HPLC fraction was lyophilized, and Cerenkov counts were measured. Based on Cerenkov counts related to ^32^P peaks, HPLC fractions were combined in alkaline polyacrylamide gel sample buffer (0.125 M Tris, pH 6.8, 6 M urea, 0.01% bromphenol blue prepared fresh) to yield 10-13 sample groups. Individual phosphorylated tryptic peptides were separated on 40% alkaline polyacrylamide gels [26]. The gel was dried and autoradiographed for 2-10 days.

### Modified manual Edman degradation

A modified version of manual Edman degradation was performed on phosphopeptides using the Millipore (Billerica, MA) Sequelon-AA acrylamine disks as previously described by this laboratory [28]47). Briefly, manual Edman degradation was performed on phosphopeptides isolated from either alkaline polyacrylamide gel slices or directly from HPLC fractions. Phosphopeptides gel slices from alkaline polyacrylamide gels were washed in 50% methanol and the peptides extracted overnight in HPLC water. The extracted peptides were lyophilized, Cerenkov counts measured, and the phosphopeptides were resuspended in 30 μl 50% acetonitrile/0.1 TFA. For HPLC fractions, the lyophilized fractions were directly resuspended in 30 μl 50% acetonitrile/0.1 TFA.

A mylar membrane was placed on a 55°C dry bath and peptides were spotted on Sequelon-AA disks and disks were dried for 15 min. Peptides were coupled to membranes by adding 5 μl carbimide (10 mg/ml) for 30 minutes at room temperature. Membranes were sequentially washed in HPLC H_2_O, 100% TFA, and 100% methanol. Phenylisothiocyanate was coupled to the N-terminus of immobilized phosphopeptides to sensitize the peptides to N-terminal cleavage. Disks were washed in methanol, dried by SpeedVac, and incubated in 0.5 ml TFA for 6 min at 55°C. The TFA solution was collected and the disks washed with 1 ml 42.5% TFA. The fractions were combined and Cerenkov counts determined as a measure of release of the phosphoamino acid residues within the peptide.

### Phosphoamino acid analysis

Phosphoamino acid analysis has been described in detail previously by this laboratory [28,51]. Isolation of phosphopeptides by HPLC and 40% alkaline acrylamide gels is described above. Lyophilized phosphopeptides were resuspended in 100-200 μl of 6N HCl to which 4 μg each of purified phospho-serine, phospho-tyrosine, or phospho-threonine was added. Samples were incubated 1 hour at 110°C. Samples were dried by SpeedVac overnight, resuspended in 15 μl of pH 1.9 buffer (2.5% formic acid, 1.8% glacial acetic acid), and spotted on 20 × 20 cm cellulose thin layer chromatography plates. Samples were electrophoresed in the first dimension for 20 min at 1500 V (toward negatively charged electrode) using the Hunter HTLE 7000 electrophoresis system (CBS Scientific Del Mar, CA). After drying, plates were then subjected to second dimension electrophoresis. Plates were equilibrated in pH 3.5 buffer (5% glacial acetic acid, 0.5% pyridine in dH_2_O) and a second round of electrophoresis was performed in pH 3.5 buffer for 15 minutes at 1300V with plates rotated 90°. Plates were dried for 1 hour. Ninhydrin (5 μg/ml acetone, Pierce, Rockford, IL) was applied to the plates using an atomizer (General Glassblowing, Inc.) and plates incubated at 80°C for 1-5 minutes to visualize phosphoamino acid standards. Standards were then outlined and radiolabeled sample phosphopeptides visualized by autoradiography. Comparison to migration of phosphoamino acid standards was used to determine the identity of phosphorylated amino acid residues.

### Generation of ERα phospho-mutants

ERα S46A, S47A S46/S47A, S282A, S294A, and S559A phospho-mutant expression plasmids were constructed using the either the Quick-change XL™ (Stratagene) or GeneEditor™ (Promega) site directed mutagenesis kit in accordance with the manufacturer's instructions. Briefly, mutagenesis primers encoding serine to alanine mutations for S47, S282, S294, and S559 were designed in accordance with Stratagene primer design software, and were as follows:

S46A-Fwd:5'-.GGTGTACCTGGACG**CCA**GCAAGCCCGCC

S46A-Rev:5'-.GGCGGGCTTGC**TGG**CGTCCAGGTACACC

S46/47A-Fwd:5'-.GGTGTACCTGGACG**CCGCAA**AGCCCGCC

Rev:5'-.GGCGGGCT**TTGCGG**CGTCCAGGTACACC

S47A- Fwd: 5'-GTGTACCTGGACAGC**GCA**AAGCCCGCCGTGTAC-3'

Rev: 5'-GTACACGGCGGGCTTTGC**GCT**GTCCAGGTACAC-3'

S282- 5'-Fwd: GGGTGAAGTGGGG**GCA**GCTGGAGACATGAGA-3'

Rev:5'-TCTCATGTCTCCAGC**TGC**CCCCACTTCACCC-3'

S294-Fwd: 5'-ACCTTTGGCCA**GCC**CCGCTCATGAT-3'

Rev: 5'-ATCATGAGCGG**GGC**CGGCCAAAGGT

S559 Fwd: 5'-GGAGGGGCAGCT**GCT**GAGGAGACG-3'

Rev:5'-CGTCTCCTCCAC**AGC**TGCCCCTCC-3'

For S47A and S282A, PCR reactions were assembled using pCR3.1wt ERα (template DNA), 125 ng each fwd and rev mutagenesis primers, 1 μl Stratagene dNTP mix, and 2.5 u *Pfu *turbo DNA polymerase. PCR conditions were as follows: 95° for 50 seconds, 60°C for 60 seconds, and 68°C for 5 minutes for 18 cycles followed by final extension for 10 minutes at 68°C. Methylated template DNA strands were digested using DPN1 and the reaction transformed into XL10 gold E. coli cells. Clones were grown on 100 μg/ml ampicillin LB agar plates and 5 selected clones were amplified in 5 ml liquid cultures with LB/amp medium. Mini-plasmid preps were performed using alkaline lysis and the isolated plasmid DNA sequenced to determine the incorporation of the desired serine to alanine mutations.

For S294A and S559A, alkaline denatured pCR3.1wt ERα (template DNA) was hybridized to mutagenic (145 ng) and antibiotic resistance oligonucleotides (2.9 ng each) and in the presence of 1× annealing buffer in 20 μl a reaction volume. The reactions were heated to 75°C, for 5 minutes, and allowed to cool to 37°C utilizing a thermal cycler. Mutant strands were synthesized in reaction utilizing 10 u T4 polymerase and 3 u DNA ligase with 1× synthesis buffer in a final volume of 30 ul, and were incubate at 37°C for 90 minutes. Plasmid DNA was subsequently transformed into BMH 71-18 mutS E. coli, cultured in the presence of GeneEditor™ antibiotic selection mix overnight, and plasmid minipreps performed. A second round of plasmid minipreps were performed using DH5α competent E. coli prior to usage in experiments. Plasmid DNA was subsequently sequenced to confirm serine to alanine mutations.

### ERα phospho-specific antibodies

Generation of phospho-specific ERα antibodies has been previously described [29]. Briefly, rabbit polyclonal antibodies to phosphorylated peptides corresponding to phosphorylated S47, S282, S294 and S559 were generated by Bethyl Laboratories (Montgomery, Texas). Immunogenic phosphopeptides were as follows:

pS47 [CEVYLDS(pS)KPAVY],

pS282, [CGRGEVG(pS)AGDMR] pS294, [CRAANLWP(pS)PLMIK] and pS559(CTSRGGA(pS)VEET).

Rabbits were injected subcutaneously with immunogen/adjuvant mixture that was re-administered at day 14 and day 44. Animal sera were collected on days 54 and 60, and subsequently at two week intervals. Antibody titers were measured by ELISA and phospho-specific antibodies were affinity purified with the corresponding antigen. Antibody specificity was validated as previously described [29]. Briefly, 10^6 ^COS-1 cells were transiently transfected with 500 ng ERα or ERα phospho-mutant expression plasmids with Fugene 6 in accordance with the manufacturer's instructions. Specificity of ERα phospho-antibodies was determined by Western immunoblotting as described below. Antibodies were further validated by *in vitro *λ phosphatase treatment [29]. 400 ng of purified baculovirus expressed ERα was incubated with 200 ng λ phosphatase in 1× phosphatase buffer for 30 minutes in a total of 200 μl at 30°C. Reactions were terminated by boiling in a final concentration of 1× Laemmli buffer for 5 minutes. The samples were then electrophoresed and immunoblotted using phospho-specific antibodies or total ERα antibodies as described below.

### Immunoprecipitation/Western blot analysis

Western blot analysis for estrogen receptor has been described previously [4]. Cells were cultured in phenol-red free growth medium supplemented with 10% charcoal stripped FBS for 24 hours prior to incubation of cells with estradiol. Cells were incubated with vehicle or 10^-8 ^estradiol for 30 minutes. Total protein was extracted in high salt buffer (10 mM Tris-HCl, pH 8; 0.4 M NaCl; 2 mM EDTA; 2 mM EGTA; 50 mM potassium phosphate; 50 mM sodium fluoride; 10 mM-mercaptoethanol; 0.1% Triton X-100; 0.2% protease inhibitor cocktail; and 0.1% phenylmethylsulfonyl fluoride) and samples denatured with Laemmli buffer with boiling for 3 minutes. Proteins were fractionated by SDS-PAGE on 10% polyacrylamide gels and electrophoretically transferred to nitrocellulose membranes for 2 hours. Blots were blocked in 5% BSA TBS-T (10 mM Tris, pH 8; 150 mM NaCl; 0.1% Tween-20), and incubated with α-ERα and α-pERα primary antibodies (1:1000) overnight at 4°C. Following washes in TBS-T, secondary detection was performed using LI-COR (Lincoln, NB) near-Infrared fluorescent labeled goat α-rabbit/α-mouse secondary antibodies (1:10000) and membranes were read using the LI-COR Odyssey infrared imaging system (Lincoln, NB). Primary antibodies used for Western Blot analysis included α-ERα (B2051, Bethyl Labs, (Montgomery, TX) or D-12, Santa Cruz Biotechnology, Santa Cruz, CA), or custom α-phospho-ERα generated at Bethyl Laboratories (Montgomery, TX) (see ERα phospho-specific antibodies).

For immunoprecipitation experiments, 10^7 ^Ishikawa, MCF7, or MCF-7-LCC2, cells were lysed in IP/Western buffer (200 mM Tris pH 8.0, 250 mM NaCl, 0.1 mM EDTA, 0.5% NP-40, 0.2% protease inhibitor cocktail; and 0.1% phenylmethylsulfonyl fluoride). 5 μg of α-ERα, pS282, pS294, or pS559 antibody and 10 μl protein A conjugated magnetic beads were added to 500 μl (2-3 mg total protein) cell lysate, incubated with turning at 4°C for 3 hours. Beads were collected using magnetic tube racks, and the beads washed 3 times with IP lysis buffer. ERα was eluted in 2× Laemmli with boiling for 3 minutes. Western blots were performed as described above.

### Luciferase reporter assay

HeLa cervical cancer cells were plated at 1 × 10^5 ^cells/well in 24 well plates in phenol red free DMEM with 10% charcoal-stripped FBS. Cells were transfected with 100 ng ERE_2_-TK-Luc reporter and 200 ng wild type ERα (wt ERα) or ERα mutant expression plasmids (S47A, S282A, S294A, or S559A) using Fugene 6 as described under tissue culture and transfections). After 24 hours, each set of transfectants were incubated with vehicle or estradiol (10^-8^M) for 18 hours. Cells were lysed with 200 μl 1× reporter lysis buffer. 100 μl was loaded and luciferase activity measured using Promega™ luciferase reporter assay system. ERα/ERα mutant expression was determined to be equivalent per unit protein, and luciferase reporter assay normalized to total protein. Each treatment group was plated in quadruplicate and the experiment repeated a minimum of three times.

### Quantitative real-time RT-PCR

Real time RT-PCR to measure pS2 and GAPDH mRNA expression was performed using the RT-PCR primers and cycling parameters described previously [4]. Briefly, HeLa cells (2 × 10^6^) cultured in phenol red free DMEM supplemented with 10% charcoal stripped FBS in 10 cm dishes were transfected with 500 ng of ERα or ERα mutant expression plasmid. 24 hours post transfection, transfectants were incubated with vehicle or estradiol (10^-8^M) for 3 hours. RNA was isolated using Trizol™ reagent according to manufacturer protocol. Single step RT-PCR was performed using iScript™ one step RT-PCR kit, BioRad™ with 500 ng RNA in 25 μl reactions. pS2 expression was normalized to GAPDH expression and expression estimated using the comparative CT method outlined in the GeneAmp 5700 users manual and as previously described by this laboratory (7). Experiments were repeated a minimum of three times.

### Statistical Analysis

In RT-PCR and reporter gene expression experiments, significance differences (P ≤ 0.05) were determined using analysis of variance (ANOVA) and Fisher's LSD post hoc analysis.

### *In vitro *kinase assay

In vitro kinase assays were performed as previously described [29,42,43,49,52,53]. Briefly, purified baculovirus expressed ERα (300 ng), (Invitrogen, Carlsbad, CA) was incubated with 100 ng recombinant human protein kinase CK2α (New England Biolabs™ Ipswich, MA) isolated from E. coli in CK2 reaction buffer (20 mM Tris-HCl 50 mM KCl 10 mM MgCl_2 _pH 7.5) supplemented with 200 μM ATP for 30 minutes at 25°C. Control reactions excluding ERα or CK2α were also assembled to assess the specificity of experimental reactions. Reactions were stopped by addition of 2× Laemmli buffer and phosphorylation was determined by Western blot analysis for total ERα and pS282 or pS559 antibodies as described above. In order to assess specificity of phosphorylation, Western blot analysis for the non-CK2 ERα phosphorylation site S118 was performed as described above. Experiments were performed a minimum of three times.

### Ethical Approval

All research performed in this study met ethical standards approved by the Tulane Office of University Research Compliance.

## Authors' contributions

CCW participated in peptide mapping experiments, mutagenesis, gene expression assays, antibody characterization, kinase activity assays, and drafted manuscript. AB participated in peptide mapping experiments, and site-directed mutagenesis of phosphorylation sites. AEG participated in peptide mapping experiments. LMC performed immunoprecipitation and immuno-assays. CLS participated in experimental design, and mutagenesis. BGR conceptualized the study, participated in the experimental design and data interpretation, and participated in drafting the manuscript. All authors have read and approved the final manuscript.

## References

[B1] Leo C Chen JD The SRC family of nuclear receptor coactivators Gene 2000 245 1 11 10.1016/S0378-1119(00)00024-X 10713439

[B2] Leo C Li H Chen JD Differential mechanisms of nuclear receptor regulation by receptor-associated coactivator 3 J Biol Chem 2000 275 5976 5982 10.1074/jbc.275.8.5976 10681591

[B3] Dutertre M Smith CL Ligand-Independent Interactions of p160/Steroid Receptor Coactivators and CREB-Binding Protein (CBP) with Estrogen Receptor-{alpha}: Regulation by Phosphorylation Sites in the A/B Region Depends on Other Receptor Domains Mol Endocrinol 2003 17 1296 1314 10.1210/me.2001-0316 12714702

[B4] Shah YM Rowan BG The Src kinase pathway promotes tamoxifen agonist action in Ishikawa endometrial cells through phosphorylation-dependent stabilization of estrogen receptor (alpha) promoter interaction and elevated steroid receptor coactivator 1 activity Mol Endocrinol 2005 19 732 748 10.1210/me.2004-0298 15528270

[B5] Bunone G Briand PA Miksicek RJ Picard D Activation of the unliganded estrogen receptor by EGF involves the MAP kinase pathway and direct phosphorylation EMBO J 1996 15 2174 2183 8641283 PMC450141

[B6] Chen D Washbrook E Sarwar N Bates GJ Pace PE Thirunuvakkarasu V Phosphorylation of human estrogen receptor alpha at serine 118 by two distinct signal transduction pathways revealed by phosphorylation-specific antisera Oncogene 2002 21 4921 4931 10.1038/sj.onc.1205420 12118371

[B7] Medunjanin S Hermani A De Servi B Grisouard J Rincke G Mayer D Glycogen synthase kinase-3 interacts with and phosphorylates estrogen receptor-alpha and is involved in the regulation of receptor activity J Biol Chem 2005 38 33006 33014 10.1074/jbc.M506758200 16076840

[B8] Joel PB Smith J Sturgill TW Fisher TL Blenis J Lannigan DA PP90(RSK1) regulates estrogen receptor-mediated transcription through phosphorylation of ser-167 Mol Cell Biol 1998 18 1978 1984 9528769 10.1128/mcb.18.4.1978 PMC121427

[B9] Arnold SF Obourn JD Jaffe H Notides AC Phosphorylation of the human estrogen receptor by mitogen-activated protein kinase and casein kinase II: consequence on DNA binding Journal of Steroid Biochemistry & Molecular Biology 1995 55 163 172 10.1016/0960-0760(95)00177-2 7495695

[B10] Arnold SF Obourn JD Jaffe H Notides AC Serine 167 is the major estradiol-induced phosphorylation site on the human estrogen receptor Mol Endocrinol 1994 8 1208 1214 10.1210/me.8.9.1208 7838153

[B11] Atsriku C Britton DJ Held JM Schilling B Scott GK Gibson BW Systematic mapping of posttranslational modifications in human estrogen receptor alpha, with emphasis on novel phosphorylation sites Mol Cell Proteomics 2008 3 467 480 10.1074/mcp.M800282-MCP200 PMC2649810 18984578

[B12] Britton DJ Scott GK Schilling B Atsriku C Held JM Gibson BW A novel serine phosphorylation site detected in the N-terminal domain of estrogen receptor isolated from human breast cancer cells J Am Soc Mass Spectrom 2008 19 729 740 10.1016/j.jasms.2008.02.008 18367407 PMC7456516

[B13] Skliris GP Rowan BG Al Dhaheri M Williams C Troup S Begic S Immunohistochemical validation of multiple phospho-specific epitopes for estrogen receptor alpha (ERalpha) in tissue microarrays of ERalpha positive human breast carcinomas Breast Cancer Res Treat 2008 3 443 353 10.1007/s10549-008-0267-z PMC3448371 19104930

[B14] Clark DE Poteet-Smith CE Smith JA Lannigan DA Rsk2 allosterically activates estrogen receptor alpha by docking to the hormone-binding domain EMBO J 2001 20 3484 3494 10.1093/emboj/20.13.3484 11432835 PMC125527

[B15] Bergqvist J Elmberger G Ohd J Linderholm B Bjohle J Hellborg H Activated ERK1/2 and phosphorylated oestrogen receptor alpha are associated with improved breast cancer survival in women treated with tamoxifen Eur J Cancer 2006 42 1104 1112 10.1016/j.ejca.2006.01.028 16603346

[B16] Murphy LC Niu Y Snell L Watson P Phospho-Serine-118 Estrogen Receptor-{alpha} Expression Is Associated with Better Disease Outcome in Women Treated with Tamoxifen Clin Cancer Res 2004 10 5902 5906 10.1158/1078-0432.CCR-04-0191 15355923

[B17] Murphy L Cherlet T Adeyinka A Niu Y Snell L Watson P Phospho-serine-118 estrogen receptor-alpha detection in human breast tumors in vivo Clin Cancer Res 2004 10 1354 1359 10.1158/1078-0432.CCR-03-0112 14977836

[B18] Gee JM Robertson JF Gutteridge E Ellis IO Pinder SE Rubini M Epidermal growth factor receptor/HER2/insulin-like growth factor receptor signalling and oestrogen receptor activity in clinical breast cancer Endocr Relat Cancer 2005 12 Suppl 1 S99 S111 10.1677/erc.1.01005 16113104

[B19] Sarwar N Kim JS Jiang J Peston D Sinnett HD Madden P Phosphorylation of ER{alpha} at serine 118 in primary breast cancer and in tamoxifen-resistant tumours is indicative of a complex role for ER{alpha} phosphorylation in breast cancer progression Endocr Relat Cancer 2006 13 851 861 10.1677/erc.1.01123 16954434

[B20] Jiang J Sarwar N Peston D Kulinskaya E Shousha S Coombes RC Phosphorylation of estrogen receptor-alpha at Ser167 is indicative of longer disease-free and overall survival in breast cancer patients Clin Cancer Res 2007 13 5769 5776 10.1158/1078-0432.CCR-07-0822 17908967

[B21] Yamashita H Nishio M Toyama T Sugiura H Kondo N Kobayashi S Low phosphorylation of estrogen receptor alpha (ERalpha) serine 118 and high phosphorylation of ERalpha serine 167 improve survival in ER-positive breast cancer Endocr Relat Cancer 2008 15 755 763 10.1677/ERC-08-0078 18550720

[B22] Holm C Kok M Michalides R Fles R Koornstra RH Wesseling J Phosphorylation of the oestrogen receptor alpha at serine 305 and prediction of tamoxifen resistance in breast cancer J Pathol 2009 217 372 379 10.1002/path.2455 18991335

[B23] Ali S Metzger D Bornert JM Chambon P Modulation of transcriptional activation by ligand-dependent phosphorylation of the human oestrogen receptor A/B region EMBO J 1993 12 1153 1160 8458328 10.1002/j.1460-2075.1993.tb05756.x PMC413317

[B24] Le Goff P Montano MM Schodin DJ Katzenellenbogen BS Phosphorylation of the human estrogen receptor. Identification of hormone-regulated sites and examination of their influence on transcriptional activity J Biol Chem 1994 269 4458 4466 8308015

[B25] Kato S Endoh H Masuhiro Y Kitamoto T Uchiyama S Sasaki H Activation of the estrogen receptor through phosphorylation by mitogen-activated protein kinase Science 1995 270 1491 1494 10.1126/science.270.5241.1491 7491495

[B26] Rowan BG Weigel NL O'Malley BW Phosphorylation of steroid receptor coactivator-1. Identification of the phosphorylation sites and phosphorylation through the mitogen-activated protein kinase pathway J Biol Chem 2000 275 4475 4483 10.1074/jbc.275.6.4475 10660621

[B27] Blom N Sicheritz-Ponten T Gupta R Gammeltoft S Brunak S Prediction of post-translational glycosylation and phosphorylation of proteins from the amino acid sequence Proteomics 2004 4 1633 1649 10.1002/pmic.200300771 15174133

[B28] Rowan BG Narayanan R Weigel NL Analysis of receptor phosphorylation Methods Enzymol 2003 364 173 202 full_text 14631846 10.1016/s0076-6879(03)64011-5

[B29] Al-Dhaheri M Rowan B Application of phosphorylation site-specific antibodies to measure nuclear receptor signaling: characterization of novel phosphoantibodies for estrogen receptor a Nuclear Receptor Signaling 2006 4 e007 10.1621/nrs.04007 16741565 PMC1472668

[B30] Brunner N Frandsen TL Holst-Hansen C Bei M Thompson EW Wakeling AE MCF7/LCC2: a 4-hydroxytamoxifen resistant human breast cancer variant that retains sensitivity to the steroidal antiestrogen ICI 182,780 Cancer Res 1993 53 3229 3232 8324732

[B31] Barkhem T Haldosen LA Gustafsson JA Nilsson S pS2 Gene expression in HepG2 cells: complex regulation through crosstalk between the estrogen receptor alpha, an estrogen-responsive element, and the activator protein 1 response element Mol Pharmacol 2002 61 1273 1283 10.1124/mol.61.6.1273 12021387

[B32] Berry M Nunez AM Chambon P Estrogen-responsive element of the human pS2 gene is an imperfectly palindromic sequence Proc Natl Acad Sci USA 1989 86 1218 1222 10.1073/pnas.86.4.1218 2919170 PMC286657

[B33] Barkhem T Haldosen LA Gustafsson JA Nilsson S Transcriptional Synergism on the pS2 Gene Promoter between a p160 Coactivator and Estrogen Receptor-alpha Depends on the Coactivator Subtype, the Type of Estrogen Response Element, and the Promoter Context Mol Endocrinol 2002 16 2571 2581 10.1210/me.2002-0051 12403846

[B34] Munstermann U Fritz G Seitz G Lu YP Schneider HR Issinger OG Casein kinase II is elevated in solid human tumours and rapidly proliferating non-neoplastic tissue Eur J Biochem 1990 189 251 257 10.1111/j.1432-1033.1990.tb15484.x 2159876

[B35] Weigel NL Moore NL Steroid receptor phosphorylation: a key modulator of multiple receptor functions Mol Endocrinol 2007 21 2311 2319 10.1210/me.2007-0101 17536004

[B36] Nichols M Rientjes JJ Stewart AF Different positioning of the ligand-binding domain helix 12 and the F domain of the estrogen receptor accounts for functional differences between agonists and antagonists EMBO J 1998 17 765 773 10.1093/emboj/17.3.765 9451001 PMC1170425

[B37] Montano MM Muller V Trobaugh A Katzenellenbogen BS The carboxy-terminal F domain of the human estrogen receptor: role in the transcriptional activity of the receptor and the effectiveness of antiestrogens as estrogen antagonists Mol Endocrinol 1995 9 814 825 10.1210/me.9.7.814 7476965

[B38] Schwartz JA Zhong L Deighton-Collins S Zhao C Skafar DF Mutations targeted to a predicted helix in the extreme carboxy-terminal region of the human estrogen receptor-alpha alter its response to estradiol and 4-hydroxytamoxifen J Biol Chem 2002 15 13202 13209 10.1074/jbc.M112215200 11823467

[B39] Koide A Zhao C Naganuma M Abrams J Deighton-Collins S Skafar DF Identification of regions within the F domain of the human estrogen receptor-alpha important for modulating transactivation and protein-protein interactions Mol Endocrinol 2007 4 829 842 10.1210/me.2006-0203 17185393

[B40] Delage-Mourroux R Martini PG Choi I Kraichely DM Hoeksema J Katzenellenbogen BS Analysis of estrogen receptor interaction with a repressor of estrogen receptor activity (REA) and the regulation of estrogen receptor transcriptional activity by REA J Biol Chem 2000 46 35848 35856 10.1074/jbc.M001327200 10960470

[B41] Lahooti H Thorsen T Aakvaag A Modulation of mouse estrogen receptor transcriptional activity by protein kinase c delta Journal of Molecular Endocrinology 1998 20 245 259 10.1677/jme.0.0200245 9584839

[B42] Tzeng DZ Klinge CM Phosphorylation of purified estradiol-liganded estrogen receptor by casein kinase II increases estrogen response element binding but does not alter ligand stability Biochemical & Biophysical Research Communications 1996 223 554 560 10.1006/bbrc.1996.0933 8687434

[B43] Landesman-Bollag E Romieu-Mourez R Song DH Sonenshein GE Cardiff RD Seldin DC Protein kinase CK2 in mammary gland tumorigenesis Oncogene 2001 20 3247 3257 10.1038/sj.onc.1204411 11423974

[B44] Yde CW Frogne T Lykkesfeldt AE Fichtner I Issinger OG Stenvang J Induction of cell death in antiestrogen resistant human breast cancer cells by the protein kinase CK2 inhibitor DMAT Cancer Lett 2007 256 229 237 10.1016/j.canlet.2007.06.010 17629615

[B45] Zhou ZX Kemppainen JA Wilson EM Identification of three proline-directed phosphorylation sites in the human androgen receptor Mol Endocrinol 1995 9 605 615 10.1210/me.9.5.605 7565807

[B46] Gioeli D Black BE Gordon V Spencer A Kesler CT Eblen ST Stress kinase signaling regulates androgen receptor phosphorylation, transcription, and localization Mol Endocrinol 2006 20 503 515 10.1210/me.2005-0351 16282370

[B47] Knotts TA Orkiszewski RS Cook RG Edwards DP Weigel NL Identification of a phosphorylation site in the hinge region of human progesterone receptor and additional amino terminal phosphorylation sites J Biol Chem 2000 11 8475 8483 10.1074/jbc.M009805200 11110801

[B48] Ahmed K Gerber DA Cochet C Joining the cell survival squad: an emerging role for protein kinase CK2 Trends Cell Biol 2002 12 226 230 10.1016/S0962-8924(02)02279-1 12062170

[B49] Bai W Rowan BG Allgood VE O'Malley BW Weigel NL Differential phosphorylation of chicken progesterone receptor in hormone-dependent and ligand-independent activation J Biol Chem 1997 272 10457 10463 10.1074/jbc.272.37.23172 9099688

[B50] Rowan BG Garrison N Weigel NL O'Malley BW 8-Bromo-cyclic AMP induces phosphorylation of two sites in SRC-1 that facilitate ligand-independent activation of the chicken progesterone receptor and are critical for functional cooperation between SRC-1 and CREB binding protein Mol Cell Biol 2000 20 8720 8730 10.1128/MCB.20.23.8720-8730.2000 11073973 PMC86491

[B51] Rowan BG Weigel NL Picard D Analysis of Steroid/Nuclear Receptor Phosphorylation The Nuclear Receptor Superfamily, A Practical Approach 1999 Oxford, UK: Oxford University Press

[B52] Bai W Weigel NL Phosphorylation and steroid hormone action. [Review] [115 refs] Vitamins & Hormones 1995 51 289 313 10.1016/S0083-6729(08)61042-0 7483325

[B53] Landesman-Bollag E Channavajhala PL Cardiff RD Seldin DC p53 deficiency and misexpression of protein kinase CK2alpha collaborate in the development of thymic lymphomas in mice Oncogene 1998 16 2965 2974 10.1038/sj.onc.1201854 9662328

